# The *Plasmodium falciparum* Translationally Controlled Tumor Protein (TCTP) Is Incorporated More Efficiently into B Cells than Its Human Homologue

**DOI:** 10.1371/journal.pone.0085514

**Published:** 2014-01-17

**Authors:** Berenice Calderón-Pérez, Beatriz Xoconostle-Cázares, Rosalía Lira-Carmona, Rosaura Hernández-Rivas, Jaime Ortega-López, Roberto Ruiz-Medrano

**Affiliations:** 1 Department of Biotechnology and Bioengineering CINVESTAV-IPN, Mexico City, Mexico; 2 Unidad de Investigación Médica en Enfermedades Infecciosas y Parasitarias, CMN Siglo XXI IMSS, México City, Mexico; 3 Department of Molecular Biomedicine, CINVESTAV-IPN, Mexico City, Mexico; Université Pierre et Marie Curie, France

## Abstract

*Plasmodium falciparum* secretes a homologue of the translationally controlled tumor protein (TCTP) into serum of infected individuals, although its role in pathogenesis or virulence is unknown. To determine the effect of *P. falciparum* TCTP on B cells as compared to human TCTP, fluorescently labeled proteins were incubated on primary cultures of mouse splenic B cells and analyzed by flow cytometry and confocal microscopy. Our results indicate that both recombinant proteins are incorporated into B cells, but differ significantly in their rate and percentage of incorporation, being significantly higher for *P. falciparum* TCTP. Furthermore, *P. falciparum* TCTP showed a lower B cell proliferative effect than human TCTP, suggesting a mechanism through which the former could interfere in the host's immune response.

## Introduction

The translationally controlled tumor protein (TCTP) is a ubiquitous protein in eukaryotes involved in several biologically relevant processes such as cell growth and reprogramming, cell cycle progression, malignant transformation, inhibition of apoptosis, cell shape and protection against stress conditions [1], [2]. Recent evidence indicates that TCTP is a central mitotic regulator in plants and animals, which underlines the functional conservation of this protein family [3].

Cytokine-like functions have been established for TCTP. Human TCTP (HsTCTP) displays an IgE-dependent histamine releasing activity in basophil cells and thus may be an important factor in triggering allergies [4]. TCTP induces the production of IL-4 and IL-13 in basophils, causing chemotaxis and IL-8 secretion from eosinophils [5], [6]. Additionally, this protein enhances B cell proliferation and increases synthesis of mainly IgM from murine splenic B cells [7]. TCTP also stimulates the secretion of IL-8 and granulocyte/macrophage colony-stimulating factor in primary cultures of human bronchial epithelial cells [8]. A homologue of TCTP with mast cell/basophil histamine-releasing activity has been described in *Plasmodium falciparum*, the causal agent of malaria. It can be found in serum of infected individuals at concentrations as high as 7 µg/mL, although its role in disease is not clear and is not always detected in such instances [9], [10]. Additionally, it has been demonstrated that recombinant *P. falciparum* TCTP (PfTCTP) *in vitro* stimulates histamine release from basophils and IL-8 secretion from eosinophils [9].

PfTCTP (Genbank Accession No. XP_001351667, PDB Accession No. 3P3K) shares 34% identity and 59% similarity with HsTCTP (Genbank Accession No. NP_003286, PDB Accession No. 1YZ1), although the structure of these proteins is quite conserved. However, a closer inspection of their structures reveals subtle differences between them. Indeed, PfTCTP three-dimensional structure revealed the presence of an extra α-helix in the segment encompassing amino acid residues 22–30, close to the proposed GTPase binding pocket [11]–[13]. These differences support the notion that PfTCTP displays an altered activity relative to its host's TCTP. Malarial TCTP could have a role in blocking the normal immune response if it displays a null or decreased B-cell activation, thus functioning as dominant negative mutant [12].

In the present study, we report the effect of recombinant PfTCTP on splenic B cell proliferation compared to HsTCTP. Our results suggest that PfTCTP induces B cell proliferation to lower levels than its human counterpart. Furthermore, B cells show an enhanced affinity for PfTCTP, thus indicating that this would effectively block the action of host TCTP. Thus, this protein could be used to further understand the mechanisms through which *Plasmodium* causes disease, as well as for the development of new strategies to control malaria.

## Materials and Methods

All animal procedures were conducted according to Institutional Laboratory Animal Care and Use Committee Guidelines. The protocol was analyzed and approved by the Bioethics Committee of the Center for Research and Advanced Studies of the National Polytechnical Institute (Protocol No. 063-12). http://picual.cinvestav.mx (Integral Administration System for the Use and Care of Laboratory Animals). Anesthesia was applied using inhaled isoflurane, 100 µg/Kg. Animals were euthanized through the use of a CO_2_ chamber, inhaled for 10 minutes and all efforts were made to minimize suffering.

### Cloning of *Plasmodium falciparum* TCTP (PfTCTP) and human TCTP (HsTCTP)

*P. falciparum* blood stage parasites from the 3D7 strain were cultured essentially as previously described [14]. Poly A+ RNA was obtained from these parasites using Oligotex Direct mRNA Mini Kit (QIAGEN) following the manufacturer's instructions. 50 ng of poly A+ RNA was reverse transcribed to cDNA with SuperScript III Reverse Transcriptase (Invitrogen) using random primers. For HsTCTP, total RNA was extracted from human peripheral blood using TRIzol (Invitrogen) according to the manufacturer's instructions. 100 ng of total RNA was reverse transcribed to cDNA with SuperScript III Reverse Transcriptase (Invitrogen) with random primers.

The coding regions of PfTCTP (GenBank Accession No. XM_001351631) and HsTCTP (GenBank Accession No. NM_003295) were obtained from the *Plasmodium* cDNA and human cDNA and by PCR using the following gene-specific primers, respectively: forward primers, PfTCTP FOR 5′-CGGGATCC **ATG**AAAGTATTTAAAGAC-3′ and HsTCTP FOR 5′-CGGGATCC **ATG**ATTATCTACCGGGAC-3′ (the BamHI restriction site is underlined and the initiation codon is in bold); reverse primers, PfTCTP REV 5′-GGAATTC **TTA**ATATTTTTCTTCAAA-3′ and HsTCTP REV 5′-GGAATTC **TTA**ACATTTTTCCATTTC-3′ (the EcoRI restriction site is underlined and the termination codon in bold). The amplified products were digested with BamHI and EcoRI enzymes (Fermentas) and cloned in frame in the pProEX HTb vector (Invitrogen) in order to be expressed fused to a 6 histidine sequence (His)_6_ for affinity purification. The correct construction was confirmed by DNA sequencing.

### Protein expression and purification

*E. coli* strain DH5α containing PfTCTP or HsTCTP in pProEX HTb was grown in 2TY medium (16 g/L tryptone, 10 g/L yeast extract, 5 g/L NaCl) with 100 µg/L ampicillin at 37°C. Protein expression was induced by the addition of 60 µM IPTG (Gold Biotechnology) at 0.7 of optical density (OD) at 600 nm and the culture was incubated for 18 h at 30°C. The cells were centrifuged, lysed by thaw–freeze cycles and disrupted by 3 cycles of sonication in 20 mM sodium phosphate (pH 7.5) based buffer. The supernatants were filtered with a 0.45-µm pore size filter, and the (His)_6_-PfTCTP and (His)_6_-HsTCTP recombinant fusion proteins were isolated using the HIS-Select Nickel Affinity Column (Sigma-Aldrich) following the manufacturer's instructions. Endotoxin was removed using a PMB-agarose column (Sigma-Aldrich) according to the method recommended by the manufacturer.

### Labeling recombinant TCTP

PfTCTP and HsTCTP recombinant purified proteins were labeled for the cell proliferation assay using an Oregon Green 488 protein labeling kit (Molecular Probes) according to the manufacturer's instructions. After the labeling reaction of 1 mg of each recombinant protein, the conjugates were purified using a purification column and the degree of labeling was determined for each protein. Additionally, 1 mg of recombinant PfTCTP was also labeled with Alexa Fluor 594 protein labeling kit (Molecular Probes) for the confocal microscopy analysis following the manufacturer's instructions.

### Isolation of splenic B cells

Splenic B cells were isolated from 4 seven-week-old BALB/c mice using the B Cell Isolation Kit (Miltenyi Biotec) by negative selection according the method suggested by the manufacturer. Red blood cells were removed by treatment with RBC lysis buffer (Sigma). Non B-cells were indirectly magnetically labeled with a cocktail of antibodies against CD43 (Ly-48), CD4 (L3T4) and Ter-119. Isolation of B cells was achieved by depletion of magnetically labeled cells. The purity of B cells (CD45R B220-positive population) was ≈95%.

### Splenic B cell proliferation assay

Isolated splenic B cells (2×10^6 ^cells/well) were plated on 6-well flat-bottom microtiter plates in RPMI 1640 medium supplemented with 10% fetal bovine serum (Gibco) and incubated with 10 µg/mL of fluorescently labeled PfTCTP or HsTCTP with Oregon Green 488 for 24–120 h at 37°C in a humidified 5% CO_2_ incubator. LPS (Sigma) at a final concentration of 1 µg/mL was used as positive control, while untreated and phosphate buffer-treated B cells were used as negative controls. Two independent experiments performed in duplicate were carried out. After each incubation time, viable B cell density was measured with an Automated Cell Counter (Invitrogen) and the incorporation of fluorescently-labeled of PfTCTP and HsTCTP into splenic B cells was analyzed by flow cytometry (FACS). A total of 20000 events/well were acquired on a FACS Calibur (Becton Dickinson, San Jose, CA) using Cell Quest Pro software. Cell cycle distributions of fluorescent-positive cells were analyzed using Cell Quest Pro software; the FACS fluorescence signal was divided into four different areas corresponding to apoptosis, G1, S and G2/M phases with respect to the DNA content, and the cells in each area were counted and expressed as the percentage of total fluorescent-positive population.

### Localization of HsTCTP and PfTCTP in B cells

To determine the localization of the recombinant proteins, isolated splenic B cells (2×10^5^ cells/well) were plated on 96-well flat bottom microtiter plates in RPMI 1640 medium supplemented with 10% fetal bovine serum (Gibco). Cells were incubated with 1 µg/mL of LPS (Sigma) for 24 h at 37°C and 5% CO_2_. After incubation, B cells were washed with PBS1x and incubated for 30 min on ice with 20 mM sodium phosphate (pH 7.5) buffer, as negative control, or 50 ng/mL of recombinant protein HsTCTP labeled with Oregon Green 488 (green) fluorochrome and/or 50 ng/mL of PfTCTP labeled with Alexa Fluor 594 (red) dye. Subsequently, cells were fixed in 4% paraformaldehyde/PBS solution for 20 min at 4°C. These were observed with a multiphotonic confocal microscope model SP5 (Leica) at 60× magnification. Images were processed and analyzed using the LAS AF software (Leica). At least 100 cells were visualized for each treatment.

### Statistical analysis

For statistical analysis of data, *p* value was analyzed using the paired Student's *t* test program (R Statistical Software 3.0.2; R Foundation for Statistical Computing, Vienna, Austria) [15]. Results were considered statistically significant when *p*<0.05.

## Results

### Effect of *P. falciparum* and human TCTP on splenic B cell proliferation

**Figure 1:**
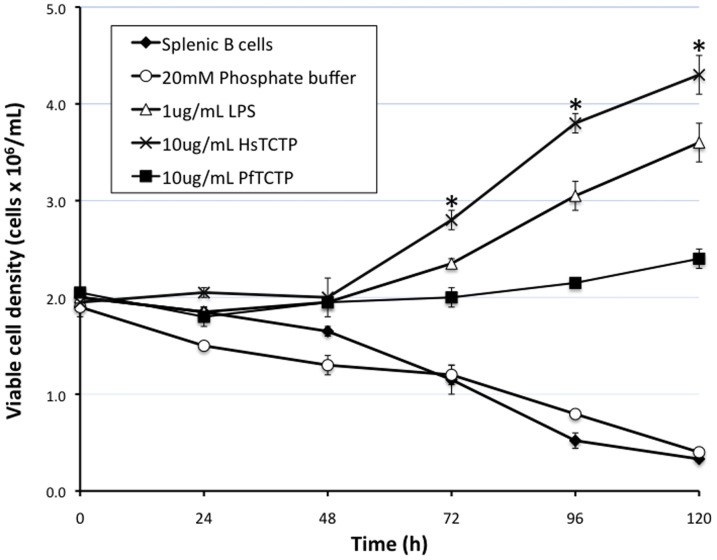
Effect of recombinant TCTP on B cell proliferation. Splenic B cells (2×10^6^cells/well) were plated with RPMI 1640 supplemented with 10% FBS and incubated with 10 µg/mL of recombinant HsTCTP or PfTCTP labeled with Oregon Green 488 (green) fluorochrome for 24–120 h at 37°C and 5% CO_2_. LPS (1 µg/mL) was used as positive control. Untreated and 20 mM phosphate buffer-treated B cells were used as negative controls. Viable B cell density was measured with a Countess Automated Cell Counter. Results are representative of two independent experiments and are expressed as the mean ±SD of duplicate cultures. *, *p*<0.05 vs 10 µg/mL labeled PfTCTP treated B cells.

The effect of PfTCTP or HsTCTP on B cells was determined by incubation of Oregon Green 488-labeled recombinant proteins on a primary culture of negatively-selected mouse splenic B cells and measurement of viable cell density with an automated cell counter. As shown in Figure 1, there is a statistical significant difference between cell density of splenic B cells treated with HsTCTP and PfTCTP from 72 h to 120 h. HsTCTP elicits the highest level of B cell proliferation after a 120 h interval. Indeed, PfTCTP, while capable of inducing proliferation, showed almost 45% lower B cell stimulatory effect than HsTCTP at 120 h. There was a significant difference between the viable B cell density elicited by HsTCTP (4.3×10^6^cells/mL ±4.5%) and PfTCTP (2.4×10^6^ cells/mL ±4.2%) recombinant proteins under the conditions tested at 120 h [*t*(1.47) = 8.49, *p* = 0.03166]. The viable cell density of untreated splenic B cells or those incubated with 20 mM phosphate buffer underwent a steady decline throughout the same period.

### Incorporation of fluorescently-labeled of PfTCTP and HsTCTP into splenic B cells

Mouse splenic B cells incubated with HsTCTP or PfTCTP fluorescently-labeled protein, as previously described, were subjected to flow cytometry to assess the incorporation of the recombinant proteins into these cells and the proportion of labeled cells in each cell cycle phase (Table 1). At 24 h, incorporation of PfTCTP into splenic B cells was already 32% higher than HsTCTP. Indeed, at 72 h and 96 h, the percentage of PfTCTP incorporated into B cells was statistically significantly different compared with the incorporation percentage of HsTCTP with *p*<0.05 and *p*<0.01, respectively. On the other hand, from 72 h to 120 h a very small increase in the percentage of incorporation of HsTCTP was observed (from 38.1% ±3.0 to 54.9% ±0.3 of total protein input). In contrast, incubated B cells with PfTCTP reached 96.4% ±0.9 of incorporation of total recombinant protein input. Regarding cell cycle progression, the percentage of fluorescently labeled B cells in each cellular phase was different during the 120 h interval. B cells labeled with HsTCTP showed a higher proportion of cells in the G1 phase at almost all time intervals assessed. In contrast, the percentage of labeled apoptotic B cells containing PfTCTP recombinant protein was higher compared to HsTCTP.

**Table 1:** Effect of recombinant TCTP in the progression of cell cycle from B cells.

Time (h)		Ap	G1	S	G2M	Incorporation
**0**	HsTCTP	0.0±0.0	81.7±3.5	8.5±1.1	9.7±2.4	**3.7**±0.3
	PfTCTP	0.0±0.0	71.3±5.3	10.9±3.3	17.8±2.1	**1.7**±0.1
**24**	HsTCTP	34.7±2.4	53.5±3.4	8.4±1.2	3.4±0.2	**22.5**±2.5
	PfTCTP	43.3±3.4	48.8±1.2	6.0±1.8	2.0±0.4	**33.0**±1.2
**48**	HsTCTP	10.8±3.3	43.5±4.8	26.1±1.5	19.6±0.1	**33.9**±1.0
	PfTCTP	35.9±4.0	37.2±3.7	20.7±1.6	6.3±1.9	**43.6**±0.8
**72**	HsTCTP	14.1±1.5	33.8±1.5	29.8±1.3	22.3±1.3	**38.1**±3.0
	PfTCTP	43.6±0.5	32.8±0.2	14.0±0.4	9.6±0.2	**52.4**±0.9
**96**	HsTCTP	26.0±2.3	46.5±2.7	13.9±0.6	13.5±1.1	**39.0**±0.7
	PfTCTP	41.4±3.4	34.6±0.3	20.1±3.7	3.8±0.1	**72.4**±2.1*
**120**	HsTCTP	50.8±4.3	37.1±3.3	8.3±0.8	3.8±0.2	**54.9**±0.3
	PfTCTP	61.3±6.9	19.3±2.2	17.0±4.0	2.4±0.7	**96.4**±0.9**

Splenic B cells (2×10^6^cells/well) were plated with RPMI 1640 supplemented with 10% FBS and incubated with 10 µg/mL of recombinant HsTCTP or PfTCTP labeled with Oregon Green 488 (green) fluorochrome for 24–120 h at 37°C and 5% CO_2_. The percentage of cell cycle distribution and incorporation of fluorescently-labeled of PfTCTP and HsTCTP into splenic B cells was analyzed by flow cytometry (FACS). Results (%) are expressed as mean ± SD of duplicate determinations. One of two similar experiments is shown. Incorporation, percentage of incorporation of fluorescently-labeled protein into B cells.

*, *p*<0.05 vs 10 µg/mL labeled HsTCTP treated B cells

**, *p*<0.01 vs 10 µg/mL labeled HsTCTP treated B cells

### Localization of HsTCTP and PfTCTP in B cells

Splenic B cells were incubated with HsTCTP labeled with Oregon Green and/or PfTCTP labeled with Alexa Fluor (red) and analyzed through confocal and bright field microscopy.

**Figure 2:**
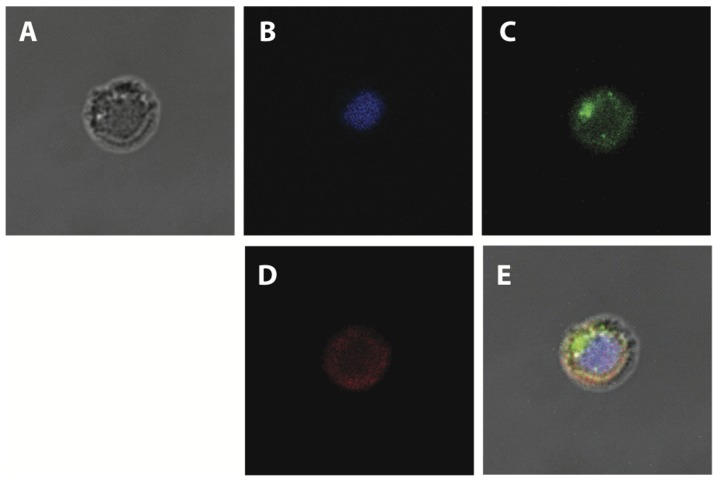
Localization of incorporated TCTP in B cells. Confocal microscopy analysis of negatively isolated B cells (2×10^5^cells/well) incubated 30 min with 50 ng/mL of recombinant HsTCTP labeled with Oregon Green 488 dye (green) and 50 ng/mL of recombinant PfTCTP labeled with Alexa Fluor 594 dye (red). Representative images are shown. A. Bright field. B. DAPI staining C. Green fluorescence. D. Red fluorescence. E. Merge.

The results shown in Figure 2 indicate that both proteins indeed attached to B cells after 30 min incubation with either of the labeled proteins. Interestingly, both are internalized into these cells quite efficiently; however, the localization of both does not overlap completely.

As can be observed in Figure 2B, fluorescent signal from HsTCTP was different compared with PfTCTP fluorescent signal when incubated simultaneously. PfTCTP showed a diffuse distribution throughout the whole cytoplasm. The images obtained were analyzed with LAS AF software (Leica) and found that the overlap correlation for both proteins range from 0.5 to 0.6 when recombinant proteins were incubated simultaneously.

## Discussion

TCTP is a central conserved regulator of growth in eukaryotes, with diverse functions in different organisms. However, the function of secreted forms of this protein is unclear and whether in multicellular eukaryotes this protein is secreted in a tissue- or cell-type-specific manner, as is likely the case in vertebrates.

PfTCTP, as well as several other forms of TCTP belonging to blood-borne parasites, are secreted to the bloodstream of its vertebrate hosts and it is reasonable to assume that it may have a role in pathogenicity and virulence [16]. However, a direct role in such processes has not been found yet. Recently, the capacity of PfTCTP to elicit histamine release and enhanced reactivity of basophils in malaria patients has been described, suggesting that it may be involved in inflammation [9], [10]. This could contribute to pathogen survival and/or spread within its host.

It has been found that indeed *Plasmodium* TCTP and human TCTP show structural variations, particularly in the domain encompassing residues 22–30 near the NH_2_ terminus, which is a β strand in HsTCTP, while in *Plasmodium knowlesi* TCTP is an α helix [12]. Intriguingly, the predicted secondary structure of TCTPs from other members of the *Plasmodium* genus suggests that this variation is conspicuous in this species (data not shown), since no β strand is predicted in this same sequence stretch, in contrast to all other TCTPs. According to a previous study in our group, the structural variation between these proteins also causes a more general structural distortion in the putative G-protein binding pocket of PfTCTP [12]. It must be mentioned that some studies have found a link between this potential structure and the function of TCTP, while others have failed to find a G-protein binding activity [17]–[20]. Nonetheless, there is sufficient evidence to suggest that the aforementioned pocket is necessary for TCTP function, although not necessarily via GTP replenishment of G-proteins.

In order to determine whether PfTCTP displays similar capabilities relative to HsTCTP that acts as B cell growth factor [7], viable B cell density and incorporation of fluorescently-labeled proteins were determined. Our results indicate that PfTCTP showed a lower B cell stimulatory effect than HsTCTP. Flow cytometry analyses revealed that both recombinant proteins are incorporated into splenic B cells, but differed significantly on the rate and percentage of incorporation. Mouse splenic B cells incorporated PfTCTP recombinant protein in a higher rate and reached 96% of protein input incorporation. According to our confocal results, both recombinant proteins are internalized within B cells, and can be accumulated in different cell compartments since PfTCTP showed a diffuse distribution throughout the whole cytoplasm.

Considering that *Plasmodium* TCTP may act like a dominant negative mutant during infection [12], our results could have interesting implications. PfTCTP is present in serum of infected individuals at high concentrations and can induce histamine release [9]; nevertheless, according to our results, it shows a lower B cell stimulatory effect compared with HsTCTP. In addition to this, splenic B cells showed a more efficient and faster incorporation of PfTCTP than HsTCTP. More detailed *in vitro* and *in vivo* assays are required to verify whether PfTCTP is implicated in preventing the onset of the immune response over an infection. It must be considered that the homology between the human and mouse TCTP is close to 96%, therefore it is probable that the latter shows similar effects and therefore the results observed in this work are biologically relevant.

TCTP lacks a signal peptide and requires a transmembrane protein for its secretion. Furthermore, a specific sequence in this protein is sufficient for its export [21]. It is not clear what fraction of the intracellular pool of TCTP is secreted, and if so, which factors trigger such release and the tissue-specificity of this process. On the other hand, there is no information regarding the putative receptor for secreted TCTP in basophils or B cells. TCTP contains a protein transduction domain (PTD) which allows for its internalization [22]. This domain is located in the NH_2_-terminus of the protein (probably amino acids 1–10). Interestingly, this domain is part of a β_strand, which in *P. knowlesi* and *P. falciparum*, but not in HsTCTP or *Schizosaccharomyces pombe*, lies partially behind the putative G-protein binding pocket [11], [12]. This structural alteration could result in both a more efficient internalization of PfTCTP and in its lower proliferative activity. Our results suggest that PfTCTP could interfere with the action of HsTCTP, although additional work is required to elucidate whether PfTCTP could block the potential receptors for HsTCTP or if cytosolic PfTCTP could interfere with the action of HsTCTP through titration of its interaction partners. It has been shown that TCTP requires dimerization in order to function as an extracellular cytokine [23], and our PfTCTP and HsTCTP preparations are most likely monomers. However, TCTP can be found in sera of healthy and asthmatic individuals as both monomers and dimers [23]. Furthermore monomers of TCTP are capable of activating B cell proliferation, so their presence in sera have probable biological significance [7].

In the present study we have found some differences on the effect of human TCTP and PfTCTP recombinant proteins on primary cultures of mouse splenic B cells. Considering that during malaria memory B cells suffer a decrease [24], [25], it is possible that PfTCTP could be also involved in this process. Our data thus also hint to a therapy targeting PfTCTP that may be helpful to control some of the effects of malaria on the host immune system. On the other hand, PfTCTP could be engineered to further enhance its ability to bind splenic B cells and, at the same time, to block proliferation. This could open new avenues to treat cancer.
